# An outbreak of norovirus gastroenteritis associated with asymptomatic food handlers in Kinmen, Taiwan

**DOI:** 10.1186/s12889-016-3046-5

**Published:** 2016-05-04

**Authors:** Meng-Yu Chen, Wan-Chin Chen, Pei-Chen Chen, Shan-Wei Hsu, Yi-Chun Lo

**Affiliations:** Office of Preventive Medicine, Centers for Disease Control, 10F, 6 Linsen S. Road, Taipei City, Taiwan; Taipei Regional Office, Centers for Disease Control, Taipei, Taiwan; Kinmen Health Department, Kinmen, Taiwan

**Keywords:** Asymptomatic food handler, Case–control study, Norovirus, Outbreak

## Abstract

**Background:**

In February 2015 an outbreak of gastroenteritis occurred in a distillery in Kinmen, Taiwan. At least 450 affected employees developed the symptoms of diarrhea and vomiting after attending a lunch banquet on 6 February. Epidemiological, laboratory and environmental investigations were conducted to identify the agent and source of this outbreak.

**Methods:**

A case–control study was carried out among lunch attendees from the distillery. Using a semi-structured questionnaire, food and beverage consumption in the lunch banquet was assessed, as well as demographic and clinical data of the exposed people. An outbreak case was defined as a diner who developed at least three following symptoms: diarrhea, vomiting, abdominal pain, nausea, chills and/or weakness in the 72 h following the lunch. Controls were defined as lunch attendees who did not have any of the above symptoms. Rectal swabs or stool samples of the symptomatic exposed diners and food handlers as well as food and environmental samples were collected to test potential bacteria and viruses. Norovirus was detected by reverse transcription-polymerase chain reaction and sequence analysis. An environmental assessment, including environmental inspection of the restaurant and a review of work practices of food workers, was undertaken.

**Results:**

Of 363 respondents with complete data, 169 met the case definition and 111 met the control definition. Consumption of pork liver in cold appetizers (adjusted odd ratio (aOR) 3.23; 95 % confidence interval (CI): 1.26–8.30) and lamb chops (aOR: 3.98, 95 % CI: 1.74–9.11) were each associated with increased risk of illness. No cases but two asymptomatic food handlers who prepared or cooked the implicated foods tested positive for norovirus genotype I.6. Food and environmental samples were negative for any bacteria. Environmental assessment indicated that hand washing facilities were not properly accessible to food handlers. Inappropriate hygiene practices in food handlers may have contributed to food contamination.

**Conclusion:**

Our investigation suggests that etiological agent of this outbreak was norovirus. The food vehicles were pork liver and lamb chops, which may have been contaminated by asymptomatic infected food handlers. Strict adherence to hand hygiene practices and access to hand washing facilities should be reinforced to prevent such foodborne outbreaks.

## Background

Norovirus is considered the major cause of acute gastroenteritis among all age groups worldwide [1–4]. The majority of human norovirus can be classified into two genogroups, I (GI) and II (GII). Norovirus is highly infectious and can be transmitted in various ways including contact with infectious individuals or contaminated environment and consumption of contaminated food. Food contamination can occur directly with human fecal matter at the source of production or with unhygienic handling by food workers excreting the virus [5]. In foodborne norovirus outbreaks for which investigators reported the source of contamination, 70 % were caused by infected food workers [6, 7].

Kinmen County is one of Taiwan’s offshore islands located off the southeastern coast of mainland China with a population of less than 128000. The main industry on the island is liquor production. On 7 February 2015 the Kinmen Health Department was notified by a local hospital of nine gastroenteritis cases among employees from a large distillery company. All had attended “Weiya”—a traditional annual event for employers to treat employees to a banquet and thank them for their hard work throughout the year—on 6 February afternoon. The lunch banquet was held in two restaurants (A and B) and attended by more than1400 employees. The lunch included ten courses and the plates were served at the table in succession by the servers in the restaurants. The food items served in the banquet were the same in two restaurants but were mainly prepared and cooked by restaurant A. Preliminary investigation by the Kinmen Health Department found that more than 450 employees of the distillery from different departments experienced gastrointestinal illness by 8 February and all affected had attended the lunch banquet. Food served at the lunch banquet was suspected as the vehicle of transmission. An investigation team from the Kinmen Health Department and Taiwan Field Epidemiology Training Program was established to identify the etiological agent and factors associated with food contamination.

## Methods

### Epidemiological investigation

List of lunch attendees and ill persons were provided by the distillery. Three departments of the distillery, consisting of 520 employees, were purposely selected and therefore lunch attendees in restaurants A and B could be included for analytic study. A semi-structured questionnaire was designed to ask about the attendee’s food and beverage consumption in the banquet, as well as demographic and clinical data. The questionnaire was distributed to all the employees from three selected departments who had attended the lunch banquet. The participants were asked to complete and return the questionnaire to the distillery by 13 February.

A case–control study was performed to determine whether certain foods were associated with illness. An outbreak case was defined as a lunch attendee reporting at least three of the following symptoms of nausea, vomiting, diarrhea or abdominal pain within 72 h of eating at the banquet. Controls were defined as lunch attendees who had not displayed any of the above symptoms during the same time period.

Data collected were analyzed using Excel, version 2010 (Microsoft) and Epi Info™ 7. We conducted univariate analyses to compare characteristics and exposure of cases and controls using the Chi-square or Fisher’s exact test for categorical variables and the Wilcoxon rank-sum test for continuous variables. All comparisons were two-tailed and a *p*-value <0.05 was considered significant. Odds ratios (OR) with 95 % confidence intervals (CI) were calculated individually for all food items and beverages. Multivariable analysis was performed using logistic regression. The multivariable model was constructed using all variables that were significantly associated with illness in univariate analysis (*P* < 0.05).

### Human microbiological investigation

Following notification of the outbreak, vomitus and rectal swabs were collected from lunch attendees with symptoms. Fresh stool samples were requested from food handlers responsible for food preparation and cooking. These specimens were sent to the Taiwan Centers for Diseases Control for bacterial and viral tests. Bacterial tests included cultures for common enteropathogens (*Salmonella* spp., *Shigella*, *Vibrio cholerae*, *Vibrio parahaemolyticus*, *Staphylococcus aureus*, *Bacillus cereus*) and toxin of *Staphylococcus aureus*. Viral tests included reverse transcription-polymerase chain reaction (RT-PCR) tests for detecting norovirus and enzyme-linked immunosorbent assay screening of rotavirus as previously described [8, 9]. Those norovirus PCR-positive specimens were then sequenced and analyzed using an ABI 3130 sequencer (Applied Biosystems, Foster City, CA, USA). Multiple nucleotide sequences were aligned and analyzed phylogenetically using MEGA 4.0 software.

### Food and environmental investigations

Health officers from the Kinmen Health Department inspected the two restaurants on 8 February. Environmental sampling using swabs was performed on food preparation surfaces to detect contamination. Food sampling was performed on the leftovers obtained from the restaurant A. Environmental surface and food samples were sent to the Taiwan Food and Drug Administration and tested for *Salmonella* spp., *Bacillus cereus*, *Vibrio parahaemolyticus*, *pathogenic Escherichia coli* (*E.coli*), *Staphylococcus aureus* and toxin of *Staphylococcus aureus*. Water samples collected from the kitchen of restaurant A were tested for total coliforms and fecal coliforms (*E. coli*).

The investigation team undertook another environmental assessment on 11 February, which included a detailed examination of menus, food items, preparation methods, food hygiene practices and work assignments. Food storage facilities, food preparation, kitchen facilities, water supply and toilets were also inspected. The attendance record of staff members was reviewed to identify any sick leave before or after the banquet.

## Results

### Epidemiological investigation

Questionnaires were returned from 376 attendees, out of total 464 questionnaires distributed (response rate 81 %). Complete data were available for 363 attendees, of which 169 persons met the case definition and 111 met the control definition. The remaining 83 respondents reported illness that did not meet the case definition and were excluded from the analysis.

**Fig. 1:**
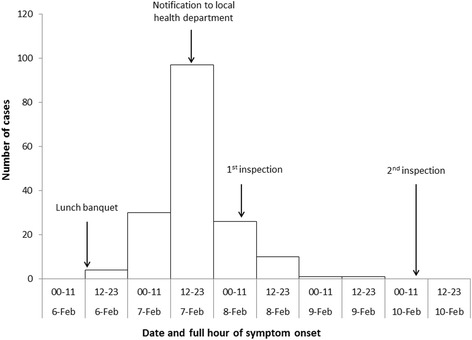
Cases of outbreak of norovirus gastroenteritis among distillery lunch attendees, by date and hour of symptom onset in 12-h interval (*n* = 169)

For both cases and controls the median age was 41 years (range: 23–65 in cases and 26–76 in controls) (*P* = 0.83). The proportion of male was significantly lower among cases than controls (57 % vs. 75 %, *P* = 0.004). Of 169 case patients, 115 (68 %) ate lunch in the restaurant B, compared with 35 (32 %) of 111 control subjects (*P* < 0.001). The most frequently reported symptoms among cases were diarrhea (71 %) and abdominal pain (62 %), followed by vomiting (55 %), nausea (41 %) and chillness (48 %). The illness onset peaked during 12:00–23:59 on 7 February (Fig. 1). The median time between eating and illness onset was 29 h (range 4–72 h). None of these cases required hospitalization.

In univariate analyses, all foods except ‘Buddha jumping over the wall’ (a traditional Chinese dish with seafood, pork and vegetables cooked in eastern jar), Guabao (steamed sandwich), cherry tomato and sliced orange were significantly associated with illness (Table 1). Consumption of beverages was not associated with illness. In the multivariable logistic regression model controlling variables including gender, restaurants and food items showing statistical evidence (*P* < 0.05) in univariate analysis, pork liver in cold appetizers (adjusted odd ratio [aOR] 3.23; 95 % confidence interval (CI): 1.26–8.30) and lamb chops wrapped in lotus leaves (aOR: 3.98, 95 % CI: 1.74–9.11) were independently associated with illness (Table 2).

**Table 1:** Food items associated with illness by univariate analysis

Food item	Cases (*n* = 169) (%)	Controls (*n* = 111) (%)	OR (95 % CI)	*P* value
Cold appetizer—fried fish	157 (92.9)	88 (80.0)	3.27 (1.54–6.93)	0.001
Cold appetizer—salted jellyfish	130 (77.4)	68 (61.3)	2.16 (1.28–3.66)	0.004
Cold appetizer—pork liver	157 (92.9)	81 (73.0)	4.85 (2.36–9.97)	<0.001
Cold appetizer—spring roll	144 (85.7)	78 (70.3)	2.54 (1.40–4.60)	0.002
Cold appetizer—sliced fried duck	129 (77.3)	66 (59.5)	2.31 (1.37–3.90)	0.02
Buddha jumping over the wall^a^	156 (92.3)	97 (87.4)	1.73 (0.78–3.84)	0.17
Steamed rice cake with shrimps	159 (94.1)	94 (84.7)	2.88 (1.26–6.54)	0.01
Stewed bamboo shoot	161 (95.8)	98 (88.3)	3.05 (1.18–7.91)	0.02
Steamed fish	147 (87.5)	83 (74.8)	2.36 (1.26–4.42)	0.006
Lamb chops, carrots, radish wrapped in lotus leaves	141 (83.9)	65 (59.1)	3.62 (2.06–6.33)	<0.001
Salted boiled shrimps	145 (86.3)	72 (64.9)	3.41 (1.90–6.15)	<0.001
Braised pork	130 (78.3)	66 (60.0)	2.41 (1.42–4.09)	0.001
Guabao (steamed sandwich)	141 (83.4)	83 (74.8)	1.70 (0.94–3.06)	0.08
Coriander	92 (54.8)	45 (40.5)	1.78 (1.09–2.89)	0.02
Mashed garlic	100 (59.5)	46 (41.4)	2.08 (1.28–3.38)	0.003
Rib soup with Chinese yam	132 (78.6)	73 (65.8)	1.91 (1.11–3.27)	0.02
Butter Swiss roll	120 (71.4)	64 (57.7)	1.84 (1.11–3.04)	0.02
Cherry tomato	92 (54.4)	49 (44.1)	1.51 (0.93–2.45)	0.09
Sliced orange	96 (57.1)	50 (45.1)	1.63 (1.00–2.64)	0.05

OR, odds ratio; CI, confidence interval

^a^a traditional Chinese dish with seafood, pork and vegetables cooked in eastern jar

**Table 2:** Food items associated with illness by multivariable logistic regression analysis

Food item	Adjusted OR* (95 % CI)	*P* value
Cold appetizer—pork liver	3.23 (1.26–8.30)	0.01
Lamb chops, carrots, radish wrapped in lotus leaves	3.98 (1.74–9.11)	0.001

OR, odds ratio; CI, confidence interval

*adjusted for food items showing statistical evidence (at *P* < 0.05) on univariate analysis, genders and restaurants

### Human microbiological investigation

Eight lunch attendees submitted one vomitus sample and seven rectal swabs. The vomitus was negative for bacteria and virus testing. All rectal swabs were negative for bacterial testing. The virological result of stool in lunch attendees was not available because fresh stool samples were not requested at the time of collection. Eight of 12 restaurant food handlers submitted fresh stool samples, of which all were negative for bacterial testing but two were positive for norovirus GI.6. These two samples were retrieved from two asymptomatic food handlers who were on duty at the lunch banquet—one responsible for cooking and cutting pork liver and the other responsible for arranging the cold appetizers including pork liver on the serving platter. The vegetables (carrots and radish) served with lamp chops were also cut by the second food handler.

### Food and environmental investigations

The initial environmental health inspection on 8 February revealed the violation of food safety regulatory requirements. Poor hygiene in the kitchen, inadequate food storage and improper waste disposal were identified. Investigation showed that kitchen staff used tap water for cooking but used untreated ground water to wash food ingredients and utensils. Subsequent sampling of tap water and ground water from the kitchen of restaurant A tested negative for total coliforms or fecal coliforms. Eleven food samples retrieved from the restaurant A were negative for bacterial testing. Four environmental samples (retrieved from chopping boards of restaurant A and B) were negative on bacterial analysis.

During the second inspection on 11 February, the investigation team found that a separate toilet was placed in the kitchen of restaurant A for staff use only but no hand wash sink was seen inside. The manager of restaurant A claimed that food workers might wash their hands using sink in the kitchen or use toilets for customers. Interview also indicated that food items were cooked 3 h before serving; some were reheated by steamers and some were not. Regarding the health status among staff before the outbreak, the manager of restaurant A denied any illness. However, reviewing the attendance records revealed that one server had taken a week’s leave to care for her child with gastroenteritis before the banquet. The two restaurants did not adhere to the regulations of annual training and medical check-ups for food workers.

### Control measures

The restaurant A was closed on 8 and 9 February for cleansing its environment. No more cases were noticed since 10 February. On 11 February, while reviewing the clean-up procedures after the incident, investigation team discovered that the restaurants had used bleach solution with inappropriate concentration. The investigation team instructed the restaurants to disinfect according to the Taiwan Centers for Disease Control guidelines for environmental cleaning and disinfection of norovirus [10]. Food workers were also asked to reinforce proper hand hygiene through washing with soap and water.

## Discussion

The outbreak of acute gastroenteritis, reported on 7 February 2015 on the distillery in Kinmen, Taiwan, was presumed to be caused by norovirus. Most of the affected persons became symptomatic within 24–48 h of having the lunch with the predominant symptoms with diarrhea, abdominal pain and vomiting. The epidemic curve of this outbreak suggested a common point source and the case–control study identified pork liver in cold appetizers and lamb chops wrapped in lotus leaves being the most likely vehicles of the transmission. Since norovirus is relatively resistant and can survive temperatures as high as 60 degrees, consumption of ready-to-eat or not thoroughly cooked foods with norovirus contamination is recognized to be high risk of infection [11].

The two asymptomatic food handlers who tested positive for norovirus were the likely source of this outbreak at the lunch. A Japanese study on norovirus-associated gastroenteritis in food-catering settings has shown high rates of norovirus infection in asymptomatic food handlers and shedding a similar number of virus particles as those who were symptomatic [12]. The presence of norovirus RNA on the hands of food handlers demonstrated that the virus could be disseminated by virus-shedding food handlers [13]. In this outbreak, it would appear that asymptomatic food handlers contaminated multiple foods during their preparation. Lack of hand sink in the kitchen toilet implied that the food handlers may not adhere to hygienic regulations, such as washing hands after toilet use. Food items which were handled manually and not heated before consumption could subsequently become contaminated from infected food handlers [11]. The time pressure resulting from the large numbers of attendees may also have contributed to a lapse in personal hygiene during preparation of meals and facilitate norovirus transmission from viral-shedding food handlers.

Food handlers have been implicated in norovirus foodborne outbreaks. In the US, 70 % foodborne norovirus outbreaks were attributed to infectious food workers [6, 14]. Symptomatic food handlers are required not to work with food until 48 h after resolution of symptoms [15]. The food handlers in this outbreak were asymptomatic. Although it was possible that food handlers did not disclose the illness honestly, they might have asymptomatic infection [16–18], which was frequently detected in food handlers with reported prevalence ranging from 1 % to 12 % [4, 12, 19–21]. In such circumstance, it would be difficult to prevent outbreaks by monitoring the health status of food handlers. Therefore, following strict hygiene practices such as proper hand washing and wearing gloves while preparing foods, especially hand-prepared and ready-to-eat foods, is crucial to minimize the risk of food contamination from food handlers with asymptomatic norovirus infection.

The environmental inspection showed no hand sink in the kitchen toilet of the restaurant. This flaw may result in suboptimal hand hygiene practices of food handlers and contamination of food. A study of food worker hand hygiene identified that hand washing was more likely to occur in restaurants with more than one hand sink and with a hand sink in the observed worker’s sight [22]. Apart from training of safety food preparation practice, multidisciplinary approaches including increased accessibility of hand washing facilities would improve food handlers’ hand hygiene compliance [23].

Our investigation has at least the following limitations. First, although the survey was done within 1 week after the banquet, we could not rule out the introduction of information bias due to the retrospective nature of the study. Second, we were unable to confirm the presence of norovirus in lunch attendees because of lack of stool specimen collection for viral study. However, predominant symptom of vomiting and incubation period of 24–48 h accord with Kaplan criteria for norovirus outbreak [24]. Detection of norovirus in food handlers suggests that the food handlers are likely the common origin of cases. Finally, food handlers might have not disclosed gastroenteritis symptoms to the investigation team because of fear of negative consequences [20]. Therefore, we could not determine whether norovirus shedding in food handlers was primarily due to asymptomatic infection. This information would have helped elucidate the association between asymptomatic excretion in food handlers and outbreaks. In-depth interviews on food handlers with reassurance that they will not suffer loss of pay if they report illness could facilitate symptom reporting.

Since norovirus outbreak can be difficult to distinguish from outbreaks of other etiologies, it is recommended to submit stool for bacterial and viral testing simultaneously so pathogen of outbreaks and chain of transmission are rapidly identified and control measures could be applied accordingly. Guidance from the Taiwan Centers for Disease Control for public health communities to collect stool specimens for foodborne outbreak was revised shortly after this outbreak [25].

## Conclusion

This outbreak was a norovirus gastroenteritis outbreak associated with asymptomatic food handlers. To reduce food contamination, strict general hygiene practices, such as thorough hand hygiene and use of gloves and protective clothing in kitchen areas should be implemented. More attention should be paid to the toilet facilities for food workers to reinforce hand hygiene practices and prevent foodborne outbreak.

### Ethics approval and consent to participate

This outbreak investigation was reviewed by Taiwan Centers for Disease Control and approved as part of the legally authorized mandate. It was therefore considered a minimal risk research, which was exempted from human subject review and does not require informed consent.

### Availability of data and materials

The dataset supporting the conclusions of this article is available in the FigShare repository https://dx.doi.org/10.6084/m9.figshare.3144727.v2.

## References

[1] Ahmed SM, Hall AJ, Robinson AE, Verhoef L, Premkumar P, Parashar UD (2014). Global prevalence of norovirus in cases of gastroenteritis: a systematic review and meta-analysis. Lancet Infect Dis.

[2] Centers for Disease Control and Prevention (2011). Updated norovirus outbreak management and disease prevention guidelines. MMWR Recomm Rep.

[3] Hall AJ, Lopman BA, Payne DC, Patel MM, Gastanaduy PA, Vinje J (2013). Norovirus disease in the United States. Emerg Infect Dis.

[4] Robilotti E, Deresinski S, Pinsky BA (2015). Norovirus. Clin Microbiol Rev.

[5] Mody RK, Griffin PM, Bennett JE, Dolin R, Blaser MJ (2014). Foodborne disease. Mandell, Douglas, and Bennett’s principles and practice of infectious diseases.

[6] Hall AJ, Wikswo ME, Pringle K, Gould LH, Parashar UD (2014). Vital signs: foodborne norovirus outbreaks - United States, 2009–2012. MMWR Morb Mortal Wkly Rep.

[7] Moe CL (2009). Preventing norovirus transmission: how should we handle food handlers?. Clin Infect Dis.

[8] Wu FT, Chen HC, Yen C, Wu CY, Katayama K, Park Y (2015). Epidemiology and molecular characteristics of norovirus GII.4 Sydney outbreaks in Taiwan, January 2012-December 2013. J Med Virol.

[9] Lai CC, Wang YH, Wu CY, Hung CH, Jiang DD, Wu FT (2013). A norovirus outbreak in a nursing home: norovirus shedding time associated with age. J Clin Virol.

[10] Centers for Disease Control (2013). Guidelines for the prevention and control of norovirus infection.

[11] Tuan Zainazor C, Hidayah MS, Chai LC, Tunung R, Ghazali FM, Son R (2010). The scenario of norovirus contamination in food and food handlers. J Microbiol Biotechnol.

[12] Ozawa K, Oka T, Takeda N, Hansman GS (2007). Norovirus infections in symptomatic and asymptomatic food handlers in Japan. J Clin Microbiol.

[13] Boxman I, Dijkman R, Verhoef L, Maat A, van Dijk G, Vennema H (2009). Norovirus on swabs taken from hands illustrate route of transmission: a case study. J Food Prot.

[14] Widdowson MA, Sulka A, Bulens SN, Beard RS, Chaves SS, Hammond R (2005). Norovirus and foodborne disease, United States, 1991–2000. Emerg Infect Dis.

[15] Food and Drug Administration (2013). Food Code 2013.

[16] Nicolay N, McDermott R, Kelly M, Gorby M, Prendergast T, Tuite G (2011). Potential role of asymptomatic kitchen food handlers during a food-borne outbreak of norovirus infection, Dublin, Ireland, March 2009. Euro Surveill.

[17] Schmid D, Kuo HW, Hell M, Kasper S, Lederer I, Mikula C (2011). Foodborne gastroenteritis outbreak in an Austrian healthcare facility caused by asymptomatic, norovirus-excreting kitchen staff. J Hosp Infect.

[18] Barrabeig I, Rovira A, Buesa J, Bartolome R, Pinto R, Prellezo H (2010). Foodborne norovirus outbreak: the role of an asymptomatic food handler. BMC Infect Dis.

[19] Jeong AY, Jeong HS, Lee JS, Park YC, Lee SH, Hwang IG (2013). Occurrence of norovirus infections in asymptomatic food handlers in South Korea. J Clin Microbiol.

[20] Yu JH, Kim NY, Lee EJ, Jeon IS (2011). Norovirus infections in asymptomatic food handlers in elementary schools without norovirus outbreaks in some regions of Incheon, Korea. J Korean Med Sci.

[21] Okabayashi T, Yokota S, Ohkoshi Y, Ohuchi H, Yoshida Y, Kikuchi M (2008). Occurrence of norovirus infections unrelated to norovirus outbreaks in an asymptomatic food handler population. J Clin Microbiol.

[22] Green LR, Radke V, Mason R, Bushnell L, Reimann DW, Mack JC (2007). Factors related to food worker hand hygiene practices. J Food Prot.

[23] Pittet D (2001). Improving adherence to hand hygiene practice: a multidisciplinary approach. Emerg Infect Dis.

[24] Kaplan JE, Feldman R, Campbell DS, Lookabaugh C, Gary GW (1982). The frequency of a Norwalk-like pattern of illness in outbreaks of acute gastroenteritis. Am J Public Health.

[25] Centers for Disease Control (2015). Manual for Infectious Specimen Collection, 2.1 edn.

